# Salvage Therapy in Acute Severe Ulcerative Colitis: Current Practice and a Look to the Future

**DOI:** 10.5152/tjg.2023.23103

**Published:** 2023-06-01

**Authors:** Natalie Tamir-Degabli, Nitsan Maharshak, Nathaniel A. Cohen

**Affiliations:** 1 Department of Gastroenterology and Liver Diseases, Tel Aviv Medical Center, Tel Aviv, Israel; 2 Tel Aviv University Sackler Faculty of Medicine, Tel Aviv, Israel

**Keywords:** Acute severe ulcerative colitis, salvage therapy, calcineurin inhibitor, infliximab, novel therapies

## Abstract

The risk of urgent bowel resection increases significantly among patients hospitalized with acute severe ulcerative colitis. In-hospital management requires quick diagnostic, therapeutic, and decision-making, combined with a multi-disciplinary approach and accessibility to multiple therapeutic options. However, the optimal strategy is still debatable. We performed a review of the current options for salvage therapy as well as novel therapy options emerging. We reviewed studies reporting outcomes of hospitalized steroid-refractory acute severe ulcerative colitis treated with salvage therapy (calcineurin inhibitors, infliximab) as well as studies using novel biologic, small molecules, antibiotics, and artificial intelligence to optimize therapy. We collected statistical data about patient factors that impact clinical management and how these can be applied to the real-life practice in order to prescribe a more personalized medicine. Several new drugs and approaches have shown benefits during the last decades for the management of acute severe ulcerative colitis. This effort is driven by the necessity of more effective, safe, and rapidly active therapeutic options with better convenient routes of administration, in order to improve therapeutic outcomes and quality of life for patients. The next step will be tailored medicine according to patients’ profiles, taking into account disease characteristics, laboratory parameters, and patients’ preferences.

**Main Points**

- Acute severe ulcerative colitis remains a medical emergency with colectomy rates during admission approaching 30%.
- Infliximab and calcineurin inhibitors are the mainstay salvage therapies in corticosteroid refractory disease.
- Although equally effective in clinical trials, there are therapy-, patient-, and care-team-related factors that influence therapeutic choice.
- Novel therapies, in particular Janus Kinase inhibitors, may provide additional options to the high-risk patients’ group
- Artificial intelligence in patient risk stratification may aid in therapeutic decision-making in the future.

## INTRODUCTION

Ulcerative colitis (UC) is a chronic, immune-mediated inflammatory disease predominantly involving the colon. The clinical presentation of UC is typically characterized by hematochezia, diarrhea, and abdominal pain. Symptoms may also include urgency and fecal incontinence, while weight loss and fever are typical features of severe disease activity. Approximately 20% of patients with UC experience at least one severe exacerbation warranting hospitalization during the course of their disease.^1^ Of these, and despite the introduction of biologic therapy, approximately 30% of patients will undergo surgery during hospitalization.^2^

Acute severe ulcerative colitis (ASUC) is diagnosed according to Truelove and Witts’ criteria and the American College of Gastroenterology Ulcerative Colitis Activity Index.^2,3^ Intravenous corticosteroids (IVCS) remain the first-line treatment for ASUC failing outpatient medical management and approximately 65% will respond.^3^ Failure to respond to IVCS is associated with a high risk of urgent bowel resection. Indeed, the Oxford criteria, defined as a CRP >45 mg/L and more than 8 daily bowel movements after 3-5 days of IVCS, indicate a risk of colectomy in 85% of such patients.^4^ As such, in those failing to respond to IVCS, medical management can be escalated to salvage therapies namely calcineurin inhibitors (CNIs) or the anti-tumor necrosis factor therapy, infliximab (IFX), with colectomy generally reserved for patients who do not respond.^3^ Although effective, there are both patient-related and disease-related factors associated with the failure of salvage therapy and challenges associated with their administration. Furthermore, there still exists a significant 20%-30% rate of treatment failure leading to colectomy.^5^ As such, the addition of multiple new therapies with novel mechanisms of action and differing modes of administration to the UC armamentarium has potentially provided other options for both salvage therapy and post-salvage maintenance.

The purpose of this article is to review salvage therapy options available for management of patients with ASUC as well as detail potential novel prognostic and therapeutic options for this high-risk patient population.

## Choice of Salvage Therapy

Traditionally, medical therapy is escalated to either IFX or a CNI (cyclosporine or tacrolimus) in patients failing to respond to IVCS.^2^ There are multiple factors that may lead to choosing one therapy over another and these will be discussed below.

### Calcineurin Inhibitor as an Induction Therapy for Acute Severe Ulcerative Colitis

Cyclosporine (CYS), a CNI, was the first successful salvage therapy with remission rates of 75%-88%;^6^ however, its side effect profile has limited its use, particularly, since the introduction of IFX as an alternative. Hypocholesterolemia and poor renal function should be excluded prior to starting treatment as these may increase serum drug levels and the risk for adverse events, in particular, neurotoxicity. Additionally, prophylaxis against *Pneumocystis jiroveci* should be initiated as combined immunosuppression increases the risk of infection.^7^ The administration is by continuous infusion with a dose between 2 mg/kg and 4 mg/kg with dosing titrated to maintain a target serum concentration of 300-400 ng/mL.^8^ In-hospitalized patients in regular wards, monitoring and maintaining this therapeutic window presents a significant drawback of this therapy. However, once the patient responds, the intravenous (IV) infusion is transitioned to an equivalent oral dose divided twice daily. Following discharge, CYS is typically continued for 1 to 3 months during which time the transition is made to maintenance therapy, which has traditionally been with thiopurines^8^ although recently more options have become available. This strategy achieves short- and long-term colectomy-free survival of about 50%.^7^ Although these success rates are promising, there still remains a significant percentage of about 30% of patients who will require colectomy at 1 year post-hospitalization.^6^

The effectiveness of CYS as a second-line salvage therapy in a patient who has failed IFX rescue therapy has also been described and may be a relevant therapeutic option in certain patients and in highly specialized centers.^9^ However, available data are limited for sequential therapy with CYS after IFX failure and the primary concern remains the safety of such a combination of immune suppressive therapies. Overall, these studies showed good response to second-line therapy and even a lower colectomy rate^10^ yet with conflicting data regarding safety profile mainly due to serious infections.

The disadvantages of CYS treatment in addition to the uncertain long-term outcomes are potential side effects and prolonged hospitalization necessitated by the IV administration of the drug. Intravenous CYS is associated with complications such as nephrotoxicity, paresthesia and seizures, hypercholesterolemia, hepatic side effects such as mild elevations in serum bilirubin, transient serum enzyme elevations (alanine transaminase and aspartate transaminase), and rarely cholestatic liver injury, infections, hyperkalemia, and hypomagnesemia,^11^ especially, with long-term exposure. Another concern with CYS therapy yet common for all CNIs is drug and food interactions due to metabolism by cytochrome (CYP) P450 enzyme. For example, grapefruit juice ingestion may increase CYS to toxic levels, and therefore, patients should be advised not to consume grapefruit or change the dosage of drugs that are affected by P450 (most commonly used are statins, PPI).^12^

Furthermore, there is often a decrease in CYS levels when transitioning from the intravenous to oral formulations and this has been shown to be associated with an increased risk of colectomy within the 3 months following discharge.^13^

In part, these factors have led to the increasing use of tacrolimus, an oral CNI, for ASUC. Despite less available publications of tacrolimus in the setting of ASUC, compared to CYS, it has shown promise in this setting with studies showing effectiveness and an acceptable safety profile compared with placebo. The initial administration dose is between 0.05 mg/kg and 0.1 mg/kg twice daily, with a target trough level between 10 and 15 ng/mL at induction. Similarly to management with CYS, upon discharge a maintenance therapy is initiated and corticosteroids are tapered. After at least 4 weeks of tacrolimus treatment, the target trough level may be decreased to 5-10 ng/mL at the discretion of the treating physician. Side effects are relatively mild and include tremor, paresthesia, headache, and electrolyte disturbances and are seen in fewer patients than those receiving CYS therapy.^14^ This ease of oral administration and the preferable safety profile makes tacrolimus a more viable option in many instances.

### Infliximab as a Salvage Therapy for Acute Severe Ulcerative Colitis

The introduction of IFX has proven a significant addition to the therapeutic options for ASUC management. In 2005, a small, but well-conducted randomized, placebo-controlled study showed the efficacy of IFX treatment for ASUC in avoiding in-hospital colectomy (29% of the patients in the IFX group underwent a colectomy compared with 67% in the placebo group, *P* = .017).^15^ Furthermore, there were no marked differences in general side effects between the groups. Subsequently, additional studies provided further support for the use of IFX as the drug of choice for salvage therapy.^16,17^

The dosing for IFX follows the standard induction and maintenance protocol with 5 mg/kg infused at weeks 0, 2, and 6 and then every 8 weeks. As IFX is a protein-based, intravenous, monoclonal antibody therapy, there are multiple factors that are thought to affect serum and tissue drug levels and potentially the therapy’s effect. These factors include low albumin levels, high or very low (~40 kg) body weight, and inflammatory burden as in elevated C-reactive protein (CRP)^18^ and should be considered prior to choosing the ideal salvage therapy and to maximize patients’ response to treatment. Further considerations should include a history of immunogenicity to other anti-TNFs or a previous exposure to IFX that can result in immunogenicity, increased risk of infusion reactions, or lack of response to treatment.^19^

These and other new insights into the pharmacokinetics of IFX have led to an interest in dose intensification in this setting.^20^ However, the evidence to support such an approach is conflicting.^21^ A meta-analysis in 2019 revealed that standard induction dosing achieves similar outcomes compared with high-dose or accelerated dosing.^22^ Furthermore, other studies have not shown the superiority of dose intensification to 10 mg/kg or dose acceleration with 5 mg/kg as such this approach cannot be routinely recommended.^23^ Nevertheless, the dosing schedules in these studies were not stratified according to the potential risk factors of IFX failure, as mentioned above. As such, studies stratifying patients by risk of IFX failure are required to determine whether there is a sub-population that may benefit from these strategies. Indeed, recently, a dashboard-guided optimized induction where dynamic and patient-specific factors were taken into account to predict appropriate dosing in order to achieve a pre-determined therapeutic drug level showed promising results, although not in the setting of ASUC.^24^ It would be interesting whether such a dashboard could be applied in the setting of ASUC to select certain patients in whom dose optimization of IFX will improve outcomes and limit over-use and cost of therapy.

### Choosing the Right Salvage Therapy

There have been a few head-to-head trials comparing the efficacy of IFX with CYS. The CONSTRUCT trial^25^ and other RCTs have demonstrated equivalent efficacy for these therapies, looking specifically at quality of life and colectomy rate within a year. In addition, the CYSIF study was a large RCT, which compared CYS vs. IFX in 115 patients with severe UC refractory to IV steroids.^26^ Results of the study showed equal effectiveness of both therapies regarding clinical response and 3-month colectomy rate. These studies highlight that other factors should also be considered when choosing the appropriate therapy, particularly in the era of increasingly personalized medicine. These factors include physician and hospital expertise and familiarity, disease severity and degree of inflammation, prior exposure to or failure of infliximab therapy, patient co-morbidities such as hypertension and renal disease, age, sex, and patient preference (Table 1).

Evaluation of disease severity in this setting refers to determining the overall inflammatory burden. At present, the best indicators of this include severe hypoalbuminemia (<3.5 g/dL), increased inflammation indices (CRP ≥25 mg/L^27^), and endoscopic severity according to the Mayo Endoscopic Score. These are in part probable surrogate markers for increased gut permeability and hence increased IFX clearance in patients with leakage of drug into the colonic lumen and fecal loss. Indeed, it has been shown that relatively high concentrations of IFX can be detected in the feces of inflammatory bowel disease (IBD) patients, especially in those with severe disease, with the highest concentrations seen in the first days following initiation of therapy.^28^

**Figure 1.**
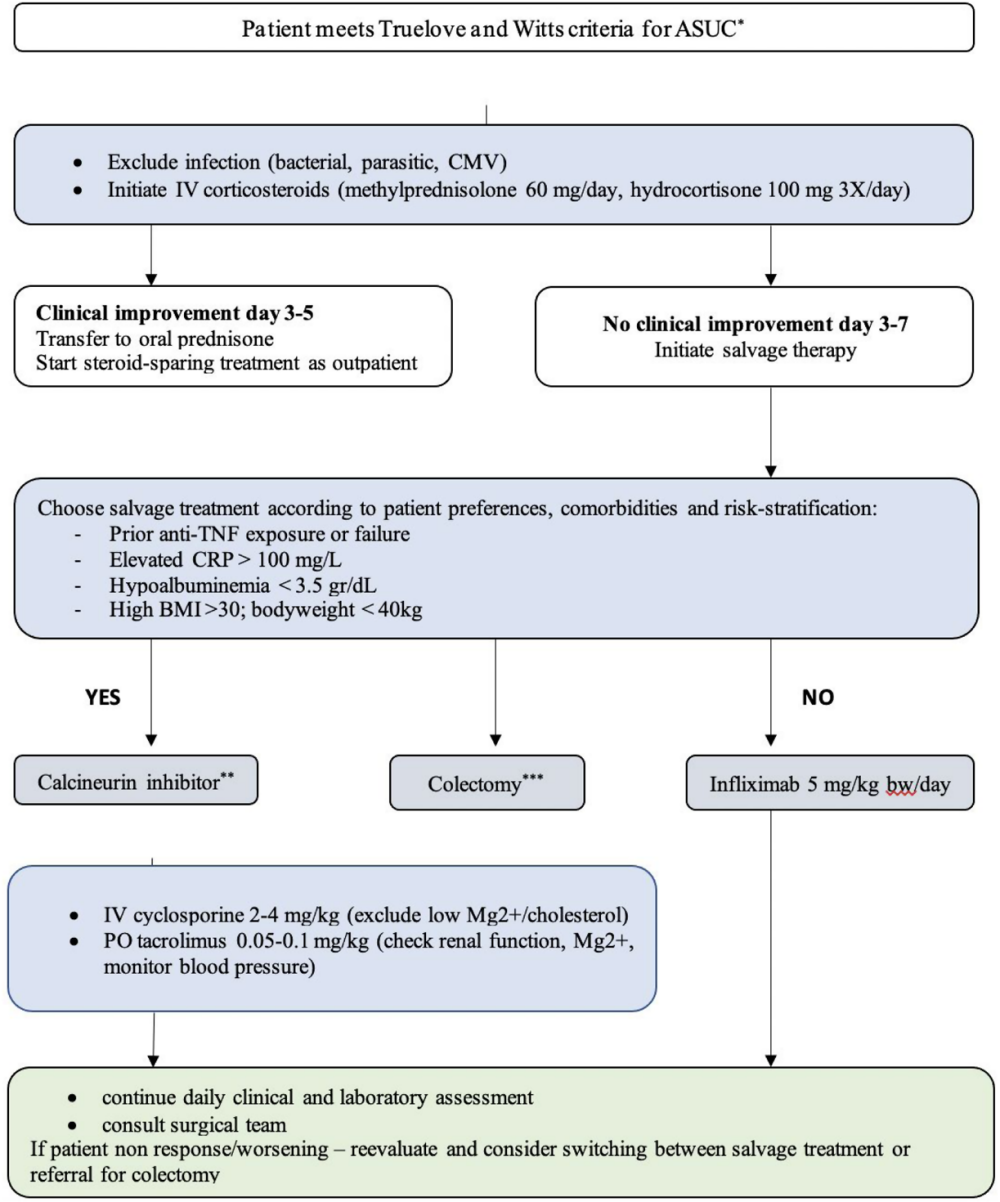
Strategy for the management of patients with acute severe ulcerative colitis. CRP, C-reactive protein; ^*^Truelove and Witts index for acute severe ulcerative colitis: frequency of bloody bowel movements ≥6/day and at least 1 feature of systemic symptoms (temperature >37.8**°**C, pulse >90 bpm, anemia ≤10.5 g/dL, erythrocyte sedimentation rate >30 mm/r). ^**^Future should consider using JAK inhibitors as an option. ^***^Colectomy should always be a part of the plan and discussed with the patients at each stage, and an early colorectal consult should always be obtained.

In addition, patient-related factors that might affect the effectiveness of the chosen treatment include elderly patients and/or co-morbidities (e.g., renal disease, uncontrolled hypertension, hypocholesterolemia), in which avoiding CNIs is preferable. In contrast, patient factors that influence the clearance rate of IFX include gender (more rapid clearance in males), body weight (more rapid clearance in high BMI but also with low body weight <40 kg)^18^, and elevated CRP^27^ and should be individually considered. A summary of stepwise clinical approach is illustrated in Figure 1.

The hospital setting also has an important role in the decision of choice of therapy. For example, the ability of the medical staff to provide and monitor a continuous infusion (administration of CYS), to perform close clinical monitoring of vital signs and adverse events, and timely monitoring of drug levels (CYS and tacrolimus) or alternatively the availability of expensive biological therapy.

Importantly, the patient should be included in the deliberations of the treatment of choice, and their preferences can be taken into consideration to the extent possible. Such an approach leads to greater cooperation and compliance which are essential in this setting.

### Colectomy as an Option for Acute Severe Ulcerative Colitis

Although medical therapy remains the primary therapeutic modality, surgery remains the “definitive” therapy for patients with longstanding UC and in particular with ASUC. As detailed above, even with optimal medical therapy, there still exists a 30% rate of colectomy in patients with ASUC. As such, a patient should always be counseled regarding this option at a relatively early stage of hospitalization (within 5-7 days). Preparation of the patient including emotional support, education, and early consultation with the surgical team is a crucial key to improving perioperative outcomes and significantly lowering in-hospital mortality and morbidity.^29^ In certain cases, direct referral for colectomy prior to initiating salvage therapy can also be considered particularly in those who were previously proven to be refractory to thiopurine or anti-TNF therapy. The initial surgery, in most cases, is a sub-total abdominal colectomy with end ileostomy. Subsequently, the patients can undergo completion proctectomy with ileal-pouch anal anastomosis (IPAA) or remain with the end ileostomy. Regarding IPAA construction, the overall outcomes are positive, with an improved quality of life and stable long-term pouch retention although potential complications should also be discussed prior to such surgery.^30^

## An Eye to the Future

The abovementioned salvage therapy strategies have been a true paradigm shift in the management of patients with ASUC. However, the remaining significant risk of treatment failure and the increasingly advanced therapy-exposed patient population requires the introduction of novel induction and maintenance therapeutic strategies for patients with ASUC.

### New Options for Maintenance Therapy after Calcineurin Inhibitors

Patients who successfully respond to CNI salvage therapy in the setting of ASUC were traditionally treated with thiopurine medications for maintenance.^31^ However, a significant proportion of patients will either be ineligible for thiopurine medications due to their thiopurine methyltransferase activity profile have previously failed or have been intolerant to thiopurine therapy. In the past, this lack of maintenance therapy options limited the use of CNIs. However, the introduction of multiple effective and safe biologic therapies to the UC armamentarium has seen a revival of CNI use. Indeed, recent studies have shown significant effectiveness and excellent safety with transitioning to vedolizumab in maintaining clinical and endoscopic response in addition to colectomy-free survival of approximately 80% at 1-year follow-up.^32^ Ustekinumab, an anti-IL12p40 antibody, has also been shown to be a suitable option for maintenance following CNI induction as detailed in the case series showing long-standing clinical remission.^33^ Ozanimod, a sphingosine-1-phosphate receptor modulator, has also been used in this setting although more data are required to examine its effectiveness.^34^ The safety of these biological and small molecule therapies is reassuring particularly considering the multiple immune-suppressive agents these patients receive during hospitalization and upon discharge. Furthermore, these options allow for the implementation of more personalized therapy strategies with intravenous, subcutaneous, and oral options allowing patients, in many instances, to choose a therapy that best suits their lifestyle and preferences.

### Novel Options for Induction in Acute Severe Ulcerative Colitis

#### JAK Inhibitors:

Tofacitinib is an oral, small-molecule JAK-1/3 inhibitor approved by the Food and Drug Administration for induction and maintenance in moderate-to-severe UC.^35^ Several characteristics make tofacitinib an attractive therapeutic option in the setting of ASUC. The drug is readily absorbed and has been shown to induce rapid clinical improvement in outpatient UC as early as day 3 of therapy.^36^ Moreover, as a small-molecule and non-protein-based therapy, tofacitinib is thought to be less susceptible to drug loss via a severely inflamed colon compared with biological medications.^37^ Additionally, JAK inhibitors are approved for the maintenance of remission and as such can be continued providing an advantage over CNI salvage therapy. These advantages have led to an interest in using tofacitinib as a salvage therapy for ASUC. In 2021, the first and largest case-control study^35^ to evaluate the efficacy and safety of JAK inhibitor therapy for ASUC showed that tofacitinib use was associated with a lower risk of colectomy at 90 days compared to controls. This benefit was most prominent in patients who received the high dose of 10 mg tofacitinib 3 times daily. Another, smaller case series retrospectively examined patients hospitalized with ASUC who were treated with a tofacitinib induction dose of 10 mg 2-3 times daily between 2019 and 2020 at 5 centers across Canada also showed effectiveness in this setting.^38^ These data have been further corroborated in other retrospective studies.^34-37,38^ The newly approved, selective JAK-1 inhibitor, upadacitinib, has been shown to improve symptoms within a day of use. These data suggest that upadacitinib may also be a promising therapeutic option for patients with ASUC.^39^ However, despite these data, there are safety concerns regarding the use of JAK inhibitors in this setting. Specifically, the reported increased risk of venous thromboembolic and cardiovascular events is concerning in this high-risk patient population. Furthermore, while the above studies are promising, they are limited by a small sample size, and RCTs are needed to fully establish the efficacy and safety of small-molecule therapy in ASUC.

#### Antibiotics Therapy:

In an effort to reduce immunosuppressive drug use, antibiotics have been explored as rescue therapies. A study in 15 pediatric patients with severe UC, most of whom were steroid-refractory or dependent, were prescribed triple antibiotic therapy with amoxicillin, metronidazole, doxycycline, and in hospitalized patients vancomycin for 2-3 weeks. Nine (60%) of the patients achieved complete remission.^40^ The use of antibiotics for patients with refractory UC was also investigated in double-blind placebo-controlled trials^41^ and resulted in improved outcomes compared to placebo.

There are a number of possible explanations as to why antimicrobial interventions are effective: targeting a specific pathogen that is associated with some of the acute UC exacerbations (i.e., *Clostridioides difficile*), translocation of bacteria due to mucosal inflammation and impairment of the epithelial barrier, and anti-inflammatory properties of some antibiotics such as ciprofloxacin. Despite a need for further prospective studies on larger populations of patients, the results suggest that antimicrobial agents might have a role to play in the treatment of exacerbations of ASUC.

#### Hyperbaric Oxygen Therapy:

Hyperbaric oxygen therapy (HBOT) increases the amount of oxygen in the plasma and tissues by breathing 100% oxygen under pressure. The high levels of oxygen created by HBOT have been found to produce a variety of downstream effects that persist well after the patient leaves the chamber. These include a reduction in pro-inflammatory cytokine production, a shift in microbiome-host metabolism, an increase in growth factor synthesis, and stem cell migration that improves wound healing.^42,43^ Considering HBOT targets several of the pathogenic mechanisms involved in UC, there is interest in using HBOT therapy in IBD in general and ASUC in particular.

Dulai et al^44^ conducted a small phase 2A trial of hyperbaric oxygen therapy for the treatment of acute moderate to severe UC flares requiring hospitalization. A total of 18 patients were enrolled and completed 5 HBOT or sham sessions. A significantly higher proportion of HBOT-treated patients achieved clinical remission compared with sham-treated patients (HBOT 50% vs. sham 0%, *P* = .05) and remission (HBOT 50% vs. sham 0%, *P *= .04). There was a >50% reduction in CRP by study day 3 in the HBOT group. A significantly lower proportion of patients required progression to second-line therapy during the hospitalization as well as those requiring colectomy as second-line therapy during hospitalization. At the 12-month follow-up, there was no difference between the 2 groups in terms of colectomy rate. The additional treatment with HBOT resulted in higher response rates and no serious side effects were reported. This unique and supplemental treatment may improve response rates in patients with ASUC. Given its favorable safety profile, HBOT may serve as an alternative to immunosuppressive medications to help bridge patients through acute UC flares; however, larger, prospective studies are required to confirm its role in this setting.^44^

#### Future Options for Improving Outcomes:

The vast array of potential data generated from genetic, mucosal, fecal, and serum samples from patients with IBD may aid in diagnosing, prognosticating, and predicting the disease’s natural history and response to therapy in a precise manner. Indeed, using machine learning techniques applied to routine clinical and laboratory data allowes for the prediction of endoscopic disease severity.^45^ Another interesting study showed that colonic miRNAs’ expression profile in patients with ASUC can classify steroid, IFX, and CYS responders versus nonresponders.^46^ Another study in patients with ASUC used AI analysis to predict response to initial medical treatment with good predictive accuracy.^47^ On the other hand, another study showed that machine learning was not superior to standard measures of disease severity at predicting response to IV steroids in patients with ASUC.^48^ The Predicting Response to Standardized Pediatric Colitis Therapy (PROTECT) study aimed to determine factors associated with response, or lack of response, to standard therapies used in the treatment of pediatric and adolescent treatment naïve patients with IBD. In that study, specific microbial genera were linked to disease severity and longitudinal microbial variations were linked to colectomy.^49^

At present, the exact role of AI, machine learning, and modeling in ASUC is unclear and is not implemented in routine clinical practice; however, there is a promise that, in the future, this will lead to a more accurate approach to classifying patients’ disease severity and likelihood to respond to a particular medication, allowing a more efficient and effective way to treat patients.^50^

## CONCLUSION

Several new drugs and approaches have been tested during the last decades for the management of ASUC. This effort is driven by the necessity of more effective and rapid therapy, with a better safety profile and convenient route administration, all of which are aimed to provide a better quality of life for patients. Moreover, despite much progress with ASUC rescue therapy, colectomy rates remain high, which indicates the constant need to improve the existing treatment options. We believe that the future is here with more advanced biologic therapies and additional options for more complex patients in the in-hospital setting. The next step will be to tailored-medicine according to patients’ profiles, taking into account disease characteristics, laboratory parameters, and patients’ preferences.

## Figures and Tables

**Table 1.** Options for Salvage Therapy Regarding Patient Profile

Factor	Example	Therapeutic Preference
Disease severity	Low albumin	Use CNI
	High CRP	Consider infliximab dose intensification
	High BMI/body weight <40 kg	Possibly JAK inhibitor
Prior therapy exposure	Infliximab	CNI (cyclosporine, tacrolimus)
Comorbidities and/or complications	Renal disease	Infliximab
	Uncontrolled hypertension	Infliximab
	Low LDL-cholesterol	Tacrolimus, infliximab
Patient-related factors		
Sex	Male	Consider infliximab dose intensification
Age	Older than 60-65	Infliximab may have a better safety profile compared with CNIs or JAK inhibitors

BMI, body mass index; CNI, calcineurin inhibitors; CRP, C-reactive protein; LDL-cholesterol, low-density cholesterol.
